# An Evaluation of the Estimated Aligners Needed to Correct Malocclusion Traits Using Invisalign ClinCheck™ Pro Software: A Retrospective Study

**DOI:** 10.3390/jcm13216552

**Published:** 2024-10-31

**Authors:** Ileana Rosa Rincon-Gregor, Cielo Ivette Bautista-Rojas, Elsy Abigail Trejo-Aké, Iván Daniel Zúñiga-Herrera, José Rubén Herrera-Atoche

**Affiliations:** 1 Department of Orthodontics, School of Dentistry, Autonomous University of Yucatan, Mérida 97000, Yucatán, Mexico; a13018495@alumnos.uady.mx (I.R.R.-G.); abigail.trejo@correo.uady.mx (E.A.T.-A.); ivan.zuniga@correo.uady.mx (I.D.Z.-H.); 2 Private Practice, Mérida 97133, Yucatán, Mexico; cieloibautista@gmail.com

**Keywords:** malocclusion, orthodontic appliances, orthodontics, corrective

## Abstract

**Background:** This study evaluated the number of aligners that Invisalign ClinCheck™ Pro Software estimates for correcting different malocclusion traits. **Methods:** This retrospective study included 157 non-extraction patients over the age of 12 years old with easy to mild malocclusions who were treated with Invisalign aligners. The Index of Complexity, Outcome, and Need (ICON) was used to evaluate the malocclusion complexity level. The number of aligners (upper, lower, and total) required to correct the malocclusion was compared based on sex, ICON level, molar and canine class, occlusal asymmetry, overbite, overjet, crowding, incisor inclination, and Bolton discrepancy. A Mann–Whitney U test (for comparisons between two groups) or a Kruskal–Wallis test (for comparisons between three or more groups) (*p* < 0.05) was used to evaluate differences in the number of aligners across variable categories. **Results:** ICON, molar class, overbite, and overjet presented significant differences (*p* < 0.05) in the number of aligners (upper, lower, and total) required to correct a malocclusion. Canine class and lower dental crowding showed significant differences in the lower and total number of aligners (*p* < 0.05). **Conclusions:** The number of aligners increases when the malocclusion presents any of the following elements: the absence of molar or canine class I, an altered overjet or overbite, severe lower crowding, or a higher complexity level. The clinician should consider these malocclusion traits when estimating the number of aligners needed for correction.

## 1. Introduction

Clear aligners were first introduced in 1997 by Invisalign™ (Align Technology, San José, CA, USA) [1]. Since then, this system has expanded its capacity to treat even more complex malocclusions. A systematic review published in 2015 concluded that clear aligners were recommended for simple cases [1]. However, Haouili (2020) [2] recently concluded that the accuracy of clear aligners has improved. The authors superimposed the initial and final ClinCheck model scans of 38 subjects to assess the accuracy of tooth movement, and they found a noticeable improvement in accuracy compared to a previous study published in 2009 [3]. According to another study, both clear aligners and braces showed the same excellent results. Using the American Board of Orthodontics Objective Grading System, the authors compared patients treated with aligners and those treated with braces. Although the treatment time was different, both groups achieved similar occlusal outcomes [4]. A survey published in 2023 concluded that most orthodontists feel comfortable treating simple and moderate cases with clear aligners. However, they avoid patients with impacted teeth or severe skeletal discrepancies [5].

In search of a way to broaden the frontiers of clear aligners, some clinical strategies have been implemented in recent years. For example, combining clear aligners with auxiliaries, such as mini-implants [5] and braces in certain treatment phases [6], has helped overcome some of the clear aligner’s limitations. In addition, Invisalign has developed new attachment designs, introduced improvements to the clear aligner materials, and incorporated the SmartStage protocol, which is a set of special instructions in its algorithm to make challenging movements more predictable [7]. Today, almost every patient is a candidate for treatment with clear aligners; however, mild to moderate malocclusion patients continue to be the main treatment focus of this system [5].

The success of clear aligners has motivated an increase in commercial options over the past two decades, and most of them offer different products based on the number of aligners needed to correct specific malocclusions. For example, Invisalign offers four products for adult patients: (1) Express (up to 7 pairs of aligners), (2) Lite (up to 14 pairs), (3) Moderate (up to 26 pairs), and (4) Comprehensive (unlimited number of aligners). Patients with high malocclusion complexity levels are easy to allocate and are usually candidates for the Comprehensive product, whereas allocation is more challenging for simpler cases. Understanding this information is important because the number of aligners determines the treatment time and cost. This study seeks to identify malocclusion traits that increase the number of aligners needed in easy to mild cases; this information will provide clinicians with a better approach to assessing treatment time and cost for these cases that are difficult to estimate.

This study evaluated the number of aligners that Invisalign ClinCheck™ Pro Software (version 6.0) estimates for correcting different malocclusion traits.

## 2. Materials and Methods

This retrospective study was carried out using a private clinic database, which exclusively uses the Invisalign system in its practice in orthodontics. All patients were treated by an orthodontist recognized as an Invisalign Diamond Doctor. Patients over 12 years of age with permanent occlusion treated with Invisalign SmartTrack material were included in the study. All patients gave informed consent to participate in the study (in the case of minors, parents or legal guardians gave their consent). Subjects affected by dental agenesis, supernumerary teeth, and/or impacted teeth were excluded. In addition, the sample did not include individuals affected by syndromes or cleft lip and/or palate. Finally, patients with extensive dental restorations were also excluded because its presence may alter the assessment of some of the study variables.

The Index of Complexity, Outcome, and Need (ICON) was used to estimate a patient’s malocclusion complexity level. The ICON classifies malocclusions as easy, mild, moderate, difficult, or very difficult to treat. The ICON assesses six components: (1) aesthetic component of the Index of Orthodontic Treatment Need (IOTN); (2) upper arch crowding/spacing; (3) crossbite; (4) vertical relationship of the incisors (open bite or overbite); and (5) buccal segment anteroposterior relationship [8]. Only easy and mild cases were included in this study.

A single operator retrieved several variables from the patients’ initial records: (1) age, (2) sex, (3) malocclusion complexity (ICON levels), (4) molar and canine class (class I or no class I), and (5) occlusal asymmetry (patients were considered to have occlusal asymmetry when they presented different molar classes on each side).

From the ClinCheck™ Pro Software (version 6.0), we obtained data on overbite (normal 0.1–4 mm, open bite < 0.1, or deep bite > 4 mm); overjet (normal 0.1–4 mm, crossbite < 0.1, or increased > 4 mm); dental crowding (no crowding/spacing; mild: up to 3 mm, moderate: >3 mm and up to 6 mm, or severe: >6 mm); incisor inclination (interincisal angle: proclined < 124°, in norm 124° to 136°, and retroclined > 136°); Bolton discrepancy (patients were divided into two groups according to the amount of dental mass discrepancy: (1) up to 2 mm and (2) greater than 2 mm); and the number of upper, lower, and total aligners (see Table 1 for the definitions of the variables). Only the first course of aligners was counted. After the patients wore the first set of aligners, the ICON level was calculated a second time, and it was compared with the initial records to evaluate the treatment results.

### 2.1. Sample Estimation

The design of this study included planned comparisons between two groups and three or more groups. The sample size estimation for comparisons of two groups was 144 patients (alpha = 0.05, power = 0.8, effect = 0.5, and ratio = 0.6), and for comparing three or more groups, it was 128 patients (alpha = 0.05, power = 0.8, effect = 0.3).

### 2.2. Error Method

The ICON value was calculated by a specially trained single operator after participating in a prior pilot test (Kappa 0.94).

### 2.3. Statistical Analysis

Using the SPSS program (version 20; IBM, Armonk, NY, USA), the Levene test was used to determine the homogeneity of variance, while the Kolmogorov–Smirnov test was applied to verify the normality of the data. The Wilcoxon test (*p* < 0.05) was used to compare the sample’s malocclusion complexity level before (T0) and after (T1) the first set of aligners was worn by the patients, and a rank-biserial correlation was utilized to estimate the effect size. The Mann–Whitney U test or Kruskal–Wallis test (*p* < 0.05) was applied to evaluate the differences in the number of aligners across the variable categories.

## 3. Results

The final sample included 157 patients, of which 38.85% were male (n = 61) and 61.14% were female (n = 96), with a mean age of 33.38 ± 12.24 years. The group’s average malocclusion complexity level was 18.9 ± 8.81. The mean number of aligners for the upper arch was 23.51 ± 10.6, 23.24 ± 11.25 for the lower arch, and 46.75 ± 20.6 for the total.

A before (T0) and after (T1) ICON comparison was possible in 137 of 157 patients (20 were eliminated because they did not have a second ClinCheck to compare with). The Wilcoxon test demonstrated that the malocclusion complexity level was significantly reduced after the first set of aligners was worn (T0 median = 18, T1 median = 12, and *p* = 0.000001), and the effect size was 0.801.

A total of 132 subjects were classified as easy cases (84.07%), and 25 were classified as mild (15.92%). The Mann–Whitney U test showed that the mild cases required more aligners (upper, lower, or total number of aligners) to correct their malocclusions than the easy ones (*p* < 0.05), as shown in Table 2.

**Figure 1:**
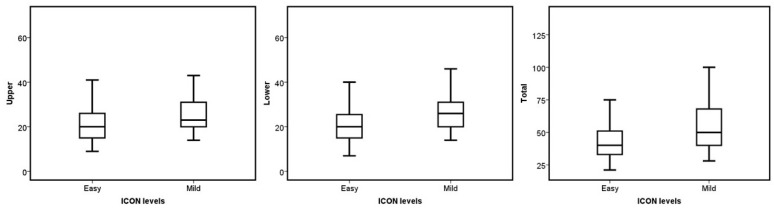
Boxplots of the upper, lower, and total aligners according to the ICON levels.

**Figure 2:**
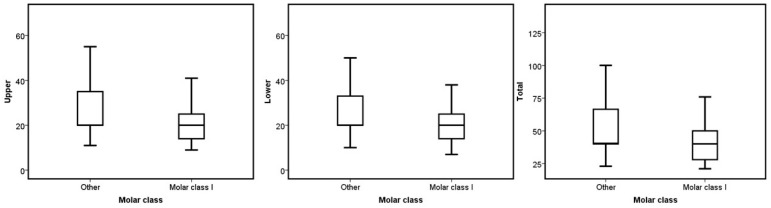
Boxplots of the upper, lower, and total aligners according to the molar class.

**Figure 3:**
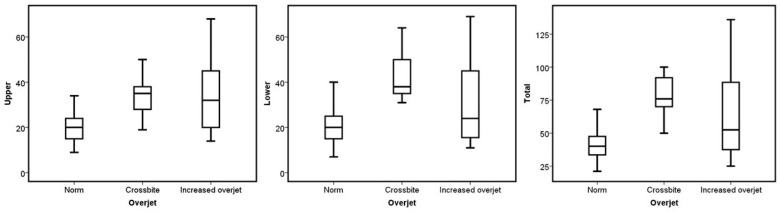
Boxplots of the upper, lower, and total aligners according to the overjet categories.

**Figure 4:**
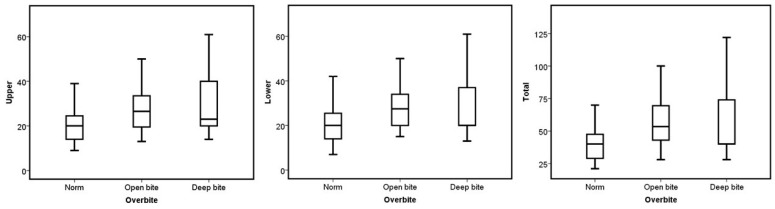
Boxplots of the upper, lower, and total aligners according to the overbite categories.

No statistical difference was found when comparing the median number of aligners based on sex, occlusal asymmetry, Bolton discrepancy, incisor angulation, and upper dental crowding (*p* > 0.05). On the other hand, ICON, molar class, overbite, and overjet presented significant differences in the upper, lower, and total number of aligners required to treat the malocclusion (*p* < 0.05). Concerning the ICON, the mild cases had a higher number of aligners in the three groups (*p* = 0.006 for the upper, *p* = 0.003 for the lower, and *p* = 0.002 for the total; see Figure 1). The cases without molar class I required more aligners for their correction (*p* = 0.014 for the upper, *p* = 0.001 for the lower, and *p* = 0.001 for the total; see Figure 2). Regarding overjet, the total number of aligners increased in patients outside the norm (*p* = 0.00007). However, in the upper arch, the number of aligners increased in the presence of augmented overjet (*p* = 0.0001), whereas in the lower arch, the number of aligners increased in subjects with crossbites (*p* = 0.001) (see Figure 3). In the case of an overbite, the upper and total aligners increased when the patient was outside of the norm (*p* = 0.0004 and *p* = 0.001, respectively); on the other hand, the lower aligners increased in open bite cases (*p* = 0.005) (see Figure 4).

**Figure 5:**
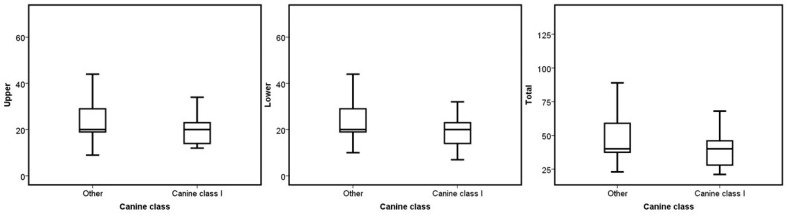
Boxplots of the upper, lower, and total aligners according to the canine class.

**Figure 6:**
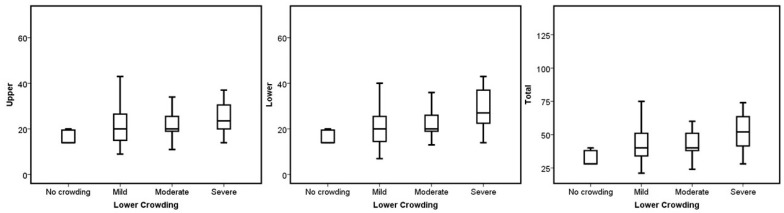
Boxplots of the upper, lower, and total aligners according to the lower dental crowding severity.

Meanwhile, canine class (*p* = 0.003 for lower and *p* = 0.015 for total; Figure 5) and lower dental crowding (*p* = 0.002 for lower and *p* = 0.016 for total; see Figure 6) presented significant differences in the lower and total number of aligners. Table 2 and Table 3 show the test results.

## 4. Discussion

The present study’s results identified malocclusion traits requiring more aligners for correction. For instance, the presence of an incisor in crossbite, a canine in a class II relationship, or severe lower crowding are indicators that a higher number of aligners and more treatment time may be needed. The comparison between the malocclusion complexity levels (mild vs. easy) showed differences, with more aligners needed to correct more complex malocclusions. Lee et al. (2023) found a similar result using the Peer Assessment Rating (PAR) index [9]. Although this similarity is expected, it is still interesting that the number of aligners needed in each category (easy or mild) presents a high standard deviation, indicating that there is a wide margin for orthodontists to consider when estimating the number of aligners needed to treat these types of malocclusions. For example, in the mild group, the average number of upper aligners was 28.16, with a standard deviation of ±11.12 (Supplementary Table S1); therefore, two-thirds of the patients classified at this level required between 17 and 39 aligners. These results demonstrate that a high dispersion of aligners is needed even in comparable malocclusions, a factor that translates into important differences in treatment time.

The results showed that the absence of class I, either on the canine or on the molar relationship, causes an increase in the number of required aligners. Class II or III malocclusions require another treatment phase to correct the molar or canine relationship. Considering that only non-extraction-treated subjects were included in our sample, inter-maxillary elastics, molar distalization, or interproximal reduction were the options for addressing the lack of a class I relationship. Scientific evidence has proven that class II malocclusions are more challenging to resolve when using clear aligners than class I [10]. In general terms, anteroposterior corrections are among the most complicated movements to achieve [11]. Some authors suggest that the sagittal correction of the molars or canines involves many concurrent movements; thus, the software prioritizes specific tooth movements over others, which compromises efficiency [10,12]. The solution to this problem is to divide the movements into different stages and not combine sagittal correction with other treatment objectives. For example, Simon et al. (2014) suggested not moving anterior teeth while distalizing molars during class II corrections [13]. Therefore, this strategy increases the number of aligners needed, no matter how mild the sagittal discrepancy is.

Concerning dental crowding, the statistical test showed mixed results when comparing severity levels. On the one hand, a statistical difference was found in the number of lower/total aligners between patients with spacing or mild crowding when compared with severe cases. On the other hand, no differences were found in the upper arch, nor between lower moderate crowding with the other groups (no crowding/spacing, mild and severe cases).

Non-extraction patients with dental crowding are usually treated with a combination of strategies, including expansion, interproximal reduction, and/or proclination [14]. Usually, mild to moderate crowding cases end with incisors in their original position (using expansion and interproximal reduction); the severe cases wind up in a proclined position [15]. Fiori et al. (2022) found that interproximal reduction (IPR) and expansion had a low, predictable value for crowding resolution, while the value for proclination was high [14]. Regarding IPR, studies have shown that clinicians do not accurately reproduce the planned amount of enamel reduction, hence the poor predictable value of IPR [14,16,17]. On the other hand, Fiori et al. (2022) found that the virtual planned expansion was not achieved, and this movement showed a wide variation [14].

Despite these results, the overall effect of combining these three strategies to solve crowding is highly efficient [14]. However, considering that only non-extraction patients with mild to moderate malocclusions were included in this study, IPR and expansion (the least predictable strategies) were the most common strategies used to solve their crowding [15]; therefore, these results are limited to subjects under similar circumstances.

Dental crowding in the lower arch was the sole variable with a transverse component, which showed statistically significant results. This is interesting because almost every clinical variable that presented no statistically significant difference with the number of aligners had a transversal component (upper crowding, Bolton discrepancy, and occlusion asymmetry). In this regard, Invisalign ClinCheck demonstrates a low predictability for movements in the transverse dimension [18,19,20,21]. Expansion is not a predictable movement because ClinCheck estimates a bodily movement of the teeth, while expansion is primarily expressed by buccolingual inclination [18,19,20,22].

On the other hand, overbite and overjet presented differences in terms of the number of aligners. This is a relevant finding because it is easy for the orthodontist to identify when a patient’s overjet or overbite is altered (crossbites, subjects with increased overjet, open bite, and deep bite).

Regarding overjet, evidence shows that ClinCheck’s incisors’ sagittal movement planning is not entirely accurate, especially when root control is required [23,24]. A large overjet also compromises the outcome [5]. When the incisor position is altered in the sagittal dimension, ClinCheck stages the correction, and more aligners are needed to achieve the desired result.

Interestingly, the total number of aligners increases regardless of whether the patient presents a crossbite or increased overjet. However, there are differences between the upper and lower arches. In the upper arch, the number of aligners increases in the presence of increased overjet, whereas in the lower arch, the number of aligners increases in subjects with crossbites. This behavior difference demonstrates how the aligners correct those malocclusions.

Concerning the vertical dimension, clear aligners correct deep bites through mandibular incisor proclination [25], upper incisor intrusion [25,26,27], and molar extrusion [25,26]. Meanwhile, open bites are corrected via incisor extrusion [25,28,29,30,31,32], retroclination [28,29,30,31,33], and, in some cases, a slight intrusion of the upper molars with mandibular counterclockwise rotation [28,30,31,32]. As can be seen, correction of the vertical dimension involves a combination of movements that cause an increase in the number of aligners. However, other studies prove that in deep bite cases, intrusion and inclination movements are not expressed as accurately as ClinCheck predicted [24]. Shahabuddin et al. found only 33% deep bite correction compared to the initial planning (2023) [34]. Thus, they suggested overcorrecting and claimed that refinements would be needed when deep bites are treated with Invisalign. Meanwhile, Al-Balaa et al. (2021) found a precision of 48.81% when comparing the predicted outcome with the actual outcome of intrusion using cone-beam computed tomography, and they proposed using auxiliaries to avoid midcourse corrections and refinements [35].

Lee et al. (2023) show that individual movements are not good predictors for the number of aligners that a malocclusion will require for correction, while a combination of movements shows the opposite results [9]. Given the many movements involved, the software stages the correction; thus, this concept may explain why altered overbite and overjet increase the number of aligners estimated for correction.

To summarize the main findings of this study, the clinician should expect that, in cases with an altered canine class, molar class, overjet, or overbite, the software will project an increased number of aligners because it stages the correction in different phases, so it would be beneficial to estimate a higher number of aligners, resulting in more time and cost to the patient.

The results of this study should be evaluated after considering its limitations. First, all subjects were treated with Invisalign, and the treatment options may vary from system to system. For example, some clear aligner systems do not recommend distalization for class II correction. Instead, they use auxiliaries, such as mini-implants. Second, even among orthodontists who use Invisalign as their clear aligner system, each orthodontist has different clinical preferences that may alter the outcome. In this case, a single orthodontist treated all subjects, so care should be taken when generalizing the results. Finally, the results of this study were limited to easy to mild malocclusions with non-extraction treatment.

Derived from these limitations, future research might be recommended. For example, it might be interesting to compare variations among different aligner systems and their results, how the orthodontists’ clinical preferences affect treatment efficiency, and whether the findings of this study can be extrapolated to moderate or severe cases.

## 5. Conclusions

According to this study’s results, clinicians should consider the absence of molar or canine class I, altered overjet or overbite, and severe lower crowding as indicators for an increase in estimated aligners needed to correct easy to mild malocclusions, and specifically in the case of Invisalign, its allocation among the different products the company offers.

The results also determined that the malocclusion complexity level affects the number of aligners required for its correction.

## Figures and Tables

**Table 1:** Definitions of the variables.

Variables	Categories	Definitions
Canine class	Canine class I	Patients with bilateral class I canine
	Other	Patients with at least one canine in a class II or III relationship
Molar class	Molar class I	Patients with bilateral class I molar
	Other	Patients with at least one molar in a class II or III relationship
Occlusal asymmetry	Symmetrical	Patients with the same molar classes on each side
	Asymmetrical	Patients with different molar classes on each side
Bolton discrepancy	Up to 2 mm of dental mass discrepancy	Up to 2 mm of dental mass discrepancy
	More than 2 mm of dental mass discrepancy	More than 2 mm of dental mass discrepancy
Overjet	Crossbite	<0.1 mm
	Norm	0.1–4 mm
	Increased overjet	>4 mm
Overbite	Open bite	<0.1 mm
	Norm	0.1–4 mm
	Deep bite	>4 mm
Incisor angulation	Proclined	<124°
	Norm	124° to 136°
	Retroclined	>136°
Crowding	No crowding	No crowding/spacing
	Mild	Up to 3 mm
	Moderate	>3 mm and up to 6 mm
	Severe	>6 mm

**Table 2:** Comparison of the number of aligners by sex, Index of Complexity, Outcome, and Need (ICON) level, canine class, molar class, occlusal asymmetry, and Bolton discrepancy based on the Mann–Whitney U test.

Variables	(N) %	Number of Aligners					
		Upper Median (IQR)	*p*	Lower Median (IQR)	*p*	Total Median (IQR)	*p*
Male	(61) 38.85%	20 (11)	0.392	20 (12.5)	0.25	40 (21.5)	0.19
Female	(96) 61.14%	20 (11)		20 (10.75)		40 (16.5)	
ICON Easy	(132) 84.07%	20 (11)	0.006 *	20 (10.75)	0.003 *	40 (18.5)	0.002 *
ICON Mild	(25) 15.92%	23 (14.5)		26 (13.5)		50 (32)	
Canine class I	(66) 42.03%	20 (9.25)	0.087	20 (9.5)	0.003 *	40 (18.25)	0.015 *
Other	(91) 57.96%	20 (10)		20 (10)		40 (23)	
Molar class I	(97) 61.78%	20 (11.5)	0.014 *	20 (11)	0.001 *	40 (22)	0.001 *
Other	(60) 38.21%	20 (15)		20 (14)		40.5 (28.25)	
Symmetrical	(129) 82.16%	20 (11)	0.945	20 (11)	0.563	40 (18.5)	0.631
Asymmetrical	(28) 17.83%	20 (5.25)		20 (5.5)		40 (11.5)	
Up to 2 mm of DMD	(120) 76.43%	20 (10)	0.258	20 (11)	0.334	40 (16.5)	0.182
More than 2 mm of DMD	(37) 23.56%	21 (10)		20 (15)		40 (25)	

(*) Statistically significant (*p* < 0.05). ICON: Index of Complexity, Outcome, and Need. DMD: Dental mass discrepancy. IQR: Interquartile range.

**Table 3:** Comparison of the number of aligners by overjet, overbite, incisor angulation, upper crowding, and lower crowding based on the Kruskal–Wallis test.

**Overjet**	**(N) %**	**Number of Aligners**					
		**Upper** ** Median (IQR)**	***p***	**Lower** ** Median (IQR)**	***p***	**Total** ** Median (IQR)**	***p***
Crossbite	(5) 3.18%	35 (20.5) ^ab^	0.0001 *	38 (24) ^b^	0.001 *	76(36) ^b^	0.00007 *
Norm	(128) 81.52%	20 (9) ^a^		20 (10) ^a^		40 (14.5) ^a^	
Increased overjet	(24) 15.29%	32 (25.5) ^b^		24 (30.5) ^ab^		52.5 (52.5) ^b^	
**Overbite**	**(N) %**	**Number of Aligners**					
		**Upper** ** Median (IQR)**	***p***	**Lower** ** Median (IQR)**	***p***	**Total** ** Median (IQR)**	***p***
Open bite	(16) 10.19%	26.5 (15) ^b^	0.0004 *	27.5 (15) ^b^	0.005 *	53.5 (30.75) ^b^	0.001 *
Norm	(116) 73.88%	20 (10.75) ^a^		20 (11.75) ^a^		40 (19.25) ^a^	
Deep bite	(25) 15.92%	23 (21.5) ^b^		20 (17.5) ^ab^		40 (37) ^b^	
**Incisor Angulation**	**(N) %**	**Number of Aligners**					
		**Upper** ** Median (IQR)**	***p***	**Lower** ** Median (IQR)**	***p***	**Total** ** Median (IQR)**	***p***
Proclined	(57) 36.3%	21 (12.5)	0.077	20 (13.5)	0.15	40 (21.5)	0.071
Norm	(56) 35.67%	20 (9)		20 (10.75)		40 (18)	
Retroclined	(44) 28.02%	20 (5.5)		20 (6)		40 (11)	
**Upper Crowding**	**(N) %**	**Number of Aligners**					
		**Upper** ** Median (IQR)**	***p***	**Lower** ** Median (IQR)**	***p***	**Total** ** Median (IQR)**	***p***
No crowding	(7) 4.46%	20 (15)	0.152	19 (10)	0.078	40 (25)	0.089
Mild	(116) 73.88%	20 (10.75)		20 (11)		40 (16.5)	
Moderate	(29) 18.47%	21 (9.5)		20 (15.5)		46 (26.5)	
Severe	(5) 3.18%	25 (12)		25 (8.5)		50 (20.5)	
**Lower Crowding**	**(N) %**	**Number of Aligners**					
		**Upper** ** Median (IQR)**	***p***	**Lower** ** Median (IQR)**	***p***	**Total** ** Median (IQR)**	***p***
No crowding	(7) 4.46%	14 (6)	0.135	14 (6) ^a^	0.002 *	28 (12) ^a^	0.016 *
Mild	(87) 55.41%	20 (12)		20 (12) ^a^		40 (19) ^a^	
Moderate	(47) 29.93%	20 (7)		20 (7) ^ab^		40 (14) ^ab^	
Severe	(16) 10.19%	23.5 (10.75)		27 (15.75) ^b^		52 (23) ^b^	

(*) Statistically significant (*p* < 0.05). IQR: Interquartile range. Different letters represent significant differences between the values.

## Data Availability

All data generated or analyzed during this study are included in this published article (and its Supplementary Materials file).

## Supplementary Materials

The following supporting information can be downloaded at: https://www.mdpi.com/article/10.3390/jcm13216552/s1, Supplementary Table S1: Variables’ descriptive statistic.

## References

[1] Rossini G., Parrini S., Castroflorio T., Deregibus A., Debernardi C.L. (2015). Efficacy of clear aligners in controlling orthodontic tooth movement: A systematic review. Angle Orthod..

[2] Haouili N., Kravitz N.D., Vaid N.R., Ferguson D.J., Makki L. (2020). Has Invisalign improved? A prospective follow-up study on the efficacy of tooth movement with Invisalign. Am. J. Orthod. Dentofac. Orthop..

[3] Kravitz N.D., Kusnoto B., BeGole E., Obrez A., Agran B. (2009). How well does Invisalign work? A prospective clinical study evaluating the efficacy of tooth movement with Invisalign. Am. J. Orthod. Dentofac. Orthop..

[4] Lin E., Julien K., Kesterke M., Buschang P.H. (2022). Differences in finished case quality between Invisalign and traditional fixed appliances: A randomized controlled trial. Angle Orthod..

[5] Abu-Arqub S., Ahmida A., Da Cunha Godoy L., Kuo C.L., Upadhyay M., Yadav S. (2023). Insight into clear aligner therapy protocols and preferences among members of the American Association of Orthodontists in the United States and Canada. Angle Orthod..

[6] Meade M.J., Weir T. (2022). A survey of orthodontic clear aligner practices among orthodontists. Am. J. Orthod. Dentofac. Orthop..

[7] Moshiri M. (2021). Product review and demonstration of the Invisalign clear aligner system. Am. J. Orthod. Dentofac. Orthop. Clin. Companion.

[8] Daniels C., Richmond S. (2000). The development of the Index of Complexity, Outcome and Need (ICON). J. Orthod..

[9] Lee S., Wu T.H., Deguchi T., Ni A., Lu W.E., Minhas S., Murphy S., Ko C.C. (2023). Assessment of malalignment factors related to Invisalign treatment time aided by automated imaging processes. Angle Orthod..

[10] Patterson B.D., Foley P.F., Ueno H., Mason S.A., Schneider P.P., Kim K.B. (2021). Class II malocclusion correction with Invisalign: Is it possible?. Am. J. Orthod. Dentofac. Orthop..

[11] Marcelino V., Baptista S., Marcelino S., Paço M., Rocha D., Gonçalves M.D.P., Azevedo R., Guimarães A.S., Fernandes G.V.O., Pinho T. (2023). Occlusal Changes with Clear Aligners and the Case Complexity Influence: A Longitudinal Cohort Clinical Study. J. Clin. Med..

[12] Taffarel I.A., Gasparello G.G., Mota-Júnior S.L., Pithon M.M., Taffarel I.P., Meira T.M., Tanaka O.M. (2022). Distalization of maxillary molars with Invisalign aligners in nonextraction patients with Class II malocclusion. Am. J. Orthod. Dentofac. Orthop..

[13] Simon M., Keilig L., Schwarze J., Jung B.A., Bourauel C. (2014). Treatment outcome and efficacy of an aligner technique—Regarding incisor torque, premolar derotation and molar distalization. BMC Oral. Health.

[14] Fiori A., Minervini G., Nucci L., d’Apuzzo F., Perillo L., Grassia V. (2022). Predictability of crowding resolution in clear aligner treatment. Prog. Orthod..

[15] Duncan L.O., Piedade L., Lekic M., Cunha R.S., Wiltshire W.A. (2016). Changes in mandibular incisor position and arch form resulting from Invisalign correction of the crowded dentition treated nonextraction. Angle Orthod..

[16] De Felice M.E., Nucci L., Fiori A., Flores-Mir C., Perillo L., Grassia V. (2020). Accuracy of interproximal enamel reduction during clear aligner treatment. Prog. Orthod..

[17] Hariharan A., Arqub S.A., Gandhi V., Da Cunha Godoy L., Kuo C.L., Uribe F. (2022). Evaluation of interproximal reduction in individual teeth, and full arch assessment in clear aligner therapy: Digital planning versus 3D model analysis after reduction. Prog. Orthod..

[18] Houle J.P., Piedade L., Todescan R., Pinheiro F.H.S.L. (2017). The predictability of transverse changes with Invisalign. Angle Orthod..

[19] Lione R., Paoloni V., Bartolommei L., Gazzani F., Meuli S., Pavoni C., Cozza P. (2021). Maxillary arch development with Invisalign system: Analysis of expansion dental movements on digital dental casts. Angle Orthod..

[20] Tien R., Patel V., Chen T., Lavrin I., Naoum S., Lee R.J., Goonewardene M.S. (2023). The predictability of expansion with Invisalign: A retrospective cohort study. Am. J. Orthod. Dentofac. Orthop..

[21] Harandi M.T., Arqub S.A., Warren E., Kuo C.L., Godoy L.D.C., Mehta S., Feldman J., Upadhyay M., Yadav S. (2023). Assessment of clear aligner accuracy of 2 clear aligners systems. Am. J. Orthod. Dentofac. Orthop..

[22] Zhou N., Guo J. (2020). Efficiency of upper arch expansion with the Invisalign system. Angle Orthod..

[23] Jiang T., Jiang Y.N., Chu F.T., Lu P.J., Tang G.H. (2021). A cone-beam computed tomographic study evaluating the efficacy of incisor movement with clear aligners: Assessment of incisor pure tipping, controlled tipping, translation, and torque. Am. J. Orthod. Dentofac. Orthop..

[24] Yan X., Zhang X., Ren L., Yang Y., Wang Q., Gao Y., Jiang Q., Jian F., Long H., Lai W. (2023). Effectiveness of clear aligners in achieving proclination and intrusion of incisors among Class II division 2 patients: A multivariate analysis. Prog. Orthod..

[25] Khosravi R., Cohanim B., Hujoel P., Daher S., Neal M., Liu W., Huang G. (2017). Management of overbite with the Invisalign appliance. Am. J. Orthod. Dentofac. Orthop..

[26] Henick D., Dayan W., Dunford R., Warunek S., Al-Jewair T. (2021). Effects of Invisalign (G5) with virtual bite ramps for skeletal deep overbite malocclusion correction in adults. Angle Orthod..

[27] Fujiyama K., Kera Y., Yujin S., Tanikawa C., Yamashiro T., Guo X., Ni A., Deguchi T. (2022). Comparison of clinical outcomes between Invisalign and conventional fixed appliance therapies in adult patients with severe deep overbite treated with nonextraction. Am. J. Orthod. Dentofac. Orthop..

[28] Harris K., Ojima K., Dan C., Upadhyay M., Alshehri A., Kuo C.L., Mu J., Uribe F., Nanda R. (2020). Evaluation of open bite closure using clear aligners: A retrospective study. Prog. Orthod..

[29] Steele B.P., Pandis N., Darendeliler M.A., Papadopoulou A.K. (2022). A comparative assessment of the dentoskeletal effects of clear aligners vs miniplate-supported posterior intrusion with fixed appliances in adult patients with anterior open bite. A multicenter, retrospective cohort study. Am. J. Orthod. Dentofac. Orthop..

[30] Suh H., Garnett B.S., Mahood K., Mahjoub N., Boyd R.L., Oh H. (2022). Treatment of anterior open bites using non-extraction clear aligner therapy in adult patients. Korean J. Orthod..

[31] Suh H., Garnett B.S., Mahood K., Boyd R.L., Oh H. (2023). Short-term stability of anterior open bite treatment with clear aligners in adults. Am. J. Orthod. Dentofac. Orthop..

[32] Blundell H.L., Weir T., Byrne G. (2023). Predictability of anterior open bite treatment with Invisalign. Am. J. Orthod. Dentofac. Orthop..

[33] Garnett B.S., Mahood K., Nguyen M., Al-Khateeb A., Liu S., Boyd R., Oh H. (2019). Cephalometric comparison of adult anterior open bite treatment using clear aligners and fixed appliances. Angle Orthod..

[34] Shahabuddin N., Kang J., Jeon H.H. (2023). Predictability of the deep overbite correction using clear aligners. Am. J. Orthod. Dentofac. Orthop..

[35] Al-Balaa M., Li H., Mohamed A.M., Xia L., Liu W., Chen Y., Omran T., Li S., Hua X. (2021). Predicted and actual outcome of anterior intrusion with Invisalign assessed with cone-beam computed tomography. Am. J. Orthod. Dentofac. Orthop..

