# Supplementary material for: An Evaluation of the Estimated Aligners Needed to Correct Malocclusion Traits Using Invisalign ClinCheck™ Pro Software: A Retrospective Study
Source: J Clin Med. 2024 Oct 31;13(21):6552. doi: 10.3390/jcm13216552 (PMC11546783; doi:10.3390/jcm13216552)
Supplement: Supplementary file 1 [file jcm-13-06552-s001.zip › jcm-3268019-supplementary.pdf]

Supplementary Table S1. Variables' descriptive statistic.

Variables	(N) %	Number of aligners		
		**Upper**	**Lower**	**Total**
		Mean SD	Mean SD	Mean SD
**Sex**				
**Male**	(61) 38.85%	24.4 ± 11.06	24.54 ± 12.51	49.16 ± 21.88
**Female**	(96) 61.14%	22.94 ± 10.32	22.28 ± 10.32	45.22 ± 19.71
**Malocclussion complexity**				
**ICON Easy**	(132) 84.07%	22.63 ± 10.31	22.25 ± 10.77	44.89 ± 20
**ICON Mild**	(25) 15.92%	28.16 ± 11.12	28.44 ± 12.46	56.6 ± 21.32
**Canine class**				
**Canine class I**	(66) 42.03%	20.98 ± 7.11	19.86 ± 7.26	40.84 ± 13
**Other**	(91) 57.96%	25.35 ± 12.26	25.69 ± 12.91	51.04 ± 23.86
**Molar class**				
**Molar class I**	(97) 61.78%	21.03 ± 7.07	20.39 ± 7.53	41.42 ± 13.51
**Other**	(60) 38.21%	27.53 ± 13.76	27.85 ± 14.4	55.38 ± 26.51
**Symmetry**				
**Symmetrical**	(129) 82.16%	23.42 ± 10.57	23.02 ± 11.11	46.44 ± 20.44
**Asymmetrical**	(28) 17.83%	23.92 ± 10.95	24.25 ± 12.01	48.17 ± 21.64
**Dental Mass Discrepancy (DMD)**				
**Up to 2 mm of DMD**	(120) 76.43%	22.75 ± 9.68	22.45 ± 10.31	45.2 ± 19.22
**More than 2 mm of DMD**	(37) 23.56%	26 ± 13.01	25.78 ± 13.71	51.78 ± 24.16
**Overjet**				
**Crossbite**	(5) 3.18%	34 ± 11.55	43.6 ± 13.42	77.6 ± 19.56
**Norm**	(128) 81.52%	21.07 ± 7.27	21.07 ± 7.52	42.14 ± 13.7
**Increased overjet**	(24) 15.29%	34.37 ± 16.35	30.58 ± 18.49	64.95 ± 33.24
**Overbite**				
**Open bite**	(16) 10.19%	28.43 ± 10.79	29.81 ± 12.97	58.25 ± 20.48
**Norm**	(116) 73.88%	21.39 ± 8.92	21.59 ± 10.1	42.99 ± 18.14
**Deep bite**	(25) 15.92%	30.2 ± 13.88	26.68 ± 13.2	56.88 ± 25.63
**Incisor angulation**				
**Proclined**	(57) 36.3%	25.91 ± 12.54	24.77 ± 13.4	50.68 ± 24.16
**Norm**	(56) 35.67%	20.58 ± 7.35	21.01 ± 8.6	41.6 ± 15.06
**Retroclined**	(44) 28.02%	24.13 ± 10.73	24.09 ± 10.96	48.22 ± 20.79
**Upper crowding**				
**No crowding**	(7) 4.46%	22.71 ± 8.93	19.14 ± 5.78	41.85 ± 11.72
**Mild**	(116) 73.88%	23.21 ± 11.09	22.46 ± 10.91	45.68 ± 21.16
**Moderate**	(29) 18.47%	24.24 ± 9.7	26.58 ± 13.51	50.82 ± 20.86
**Severe**	(5) 3.18%	27.4 ± 6.54	27.6 ± 5.54	55 ± 11.87
**Lower crowding**				
**No crowding**	(7) 4.46%	20.42 ± 12.43	16.42 ± 3.04	36.85 ± 14.69
**Mild**	(87) 55.41%	23.04 ± 10.04	22.31 ± 11.53	45.35 ± 20.42
**Moderate**	(47) 29.93%	23.68 ± 10.72	23.51 ± 9.78	47.19 ± 19.39
**Severe**	(16) 10.19%	26.93 ± 12.62	30.5 ± 13.19	57.43 ± 24.55

SD: Standard Deviation. ICON: Index of Complexity, Outcome and Need. DMD: Dental Mass Discrepancy.
